# Self-Care Practices of Patients with Non-Communicable Diseases during the COVID-19 Pandemic: A Qualitative Study

**DOI:** 10.3390/ijerph19159727

**Published:** 2022-08-07

**Authors:** Apichai Wattanapisit, Tida Sottiyotin, Jaruporn Thongruch, Sanhapan Wattanapisit, Siranee Yongpraderm, Pichawee Kowaseattapon

**Affiliations:** 1 School of Medicine, Walailak University, Thasala, Nakhon Si Thammarat 80160, Thailand; 2 Family Medicine Clinic, Walailak University Hospital, Thasala, Nakhon Si Thammarat 80160, Thailand; 3 School of Pharmacy, Walailak University, Thasala, Nakhon Si Thammarat 80160, Thailand; 4 Thasala Hospital, Thasala, Nakhon Si Thammarat 80160, Thailand

**Keywords:** self-care, non-communicable disease, COVID-19, qualitative study

## Abstract

Individuals with chronic non-communicable diseases (NCDs) have a higher risk of morbidity and mortality. This study explores the lived experience of patients with NCDs during the COVID-19 pandemic and the impact of COVID-19 on their self-care. An interpretive phenomenological analysis approach was used that involved in-depth interviews with patients who received medical services from a family medicine clinic, along with caregivers who responded on their behalf. An inductive thematic approach was utilized to analyze the data. Interview respondents included 17 patients with NCDs and four caregivers. The patients had a mean age of 65.7 ± 11.3 years and were diagnosed with an NCD, a mean of 4.8 ± 1.1 years previously. Self-care practices used during the pandemic were classified as therapeutic or preventive. Patients responded to changes in healthcare services by seeking in-person services for their acute illnesses and accepting remote services for underlying chronic conditions. The COVID-19 pandemic influenced the self-care practices of patients with NCDs. Most patients paid more attention to self-care during this time, while some became more concerned with other aspects of their life.

## 1. Introduction

As of 5 January 2022, the coronavirus disease 2019 (COVID-19) pandemic had resulted in >293 million confirmed cases and contributed to >5.4 million deaths worldwide [1]. The pandemic has affected both health and non-health sectors [2,3], and impacted several dimensions of human life [4,5,6]. In addition, public health policies and regulations relating to the pandemic, such as stay-at-home orders, social distancing, and lockdown have also influenced health and wellbeing [7].

People living with chronic non-communicable diseases (NCDs) such as diabetes mellitus, hypertension, cardiovascular diseases, chronic obstructive pulmonary disease, chronic kidney disease, and cancer are at higher risk for COVID-19 infection and more serious clinical outcomes [8]. This may be explained by particular lifestyle characteristics of patients with NCDs, including physical inactivity, tobacco smoking, and alcohol use, which may worsen the severity of COVID-19 [9].

Previous studies revealed impacts of the COVID-19 pandemic on patients with NCDs. Devi et al. reported that 19.0% of 1487 respondents had worse chronic health conditions, 61.5% experienced feelings of stress, and 50.1% felt isolated or lonely [10]. Mahmood et al. reported 32% of 401 respondents perceived themselves as more vulnerable, while 58.6% and 65.3% mentioned the negative impacts of the lockdown on their physical and mental health, respectively [11]. Moreover, COVID-19-related public health policies and regulations contributed to decreased physical activity, changes in diet, and reduced social interactions, which could worsen clinical manifestations and increase NCD comorbidities [12].

During the COVID-pandemic, service providers altered service practices, such as limiting the number of patients and providing remote consultation. These changes affect the accessibility to healthcare services and the continuity of care [12,13]. A study found that 16% of patients had a lack of medication adherence, while 31.1% missed medical appointments [10]. Fear of COVID-19 contact was stated as the most common cause of missing appointments [10].

Although the aforementioned studies indicated several effects of the COVID-19 pandemic on patients with NCDs, some aspects such as individual’s experiences and self-care practices are still unclear. To fill the gap in knowledge, this study aims to explore the lived experience of patients with NCDs during the COVID-19 pandemic and how this has impacted self-care.

## 2. Materials and Methods

### 2.1. Study Design

This qualitative study employed an interpretive phenomenological approach to explore the lived experience of patients with chronic NCDs during the COVID-19 pandemic. The interpretive phenomenology was utilized to understand and interpret the various perspectives toward lives and experiences of participants and the participants’ complex behavior patterns [14]. Relativism (reality is subjective and differs among people) was the ontological assumption, and subjectivism (knowledge is based on real-world experience) was the epistemological assumption of this study [15]. The Consolidated Criteria for Reporting Qualitative Research (COREQ) and the Standards for Reporting Qualitative Research (SRQR) were adopted to assure transparency of the qualitative methodologies and prepare the manuscript [16,17].

### 2.2. Participants and Context

The study participants included both patients and caregivers. Patients were invited if they met the following criteria: ≥18 years of age and living with chronic NCDs such as hypertension, diabetes mellitus, and hyperlipidemia who had visited the Family Medicine Clinic at Walailak University Hospital prior to December 2019 (before the emergence of COVID-19) and had at least three follow-up appointments during the pandemic. Family members of patients with NCDs who were acting as responsible caregivers were invited to add triangulation and credibility to the data [18].

During the June to November 2021 study period, the Family Medicine Clinic was operating normally but the patterns of services had been adjusted. Telemedicine (i.e., communicating by phone calls) and telepharmacy (i.e., delivering medicines from the hospital to a patient’s home) were implemented. Patients with appointments were contacted by administrative nursing staff to evaluate the necessity of in-person visits. Otherwise, patients and caregivers had the right to select whether their visits were in-person or on-call. Patients with acute or urgent medical conditions were encouraged to make an in-person visit. The hospital obtained contact history and conducted temperature screening, and required physical distancing, mask-wearing, and frequent hand washing as preventive strategies.

### 2.3. Data Collection

One author (TS), a pharmacist who had obtained a PhD in Health Social Science and was experienced in qualitative research, conducted all the in-depth interviews (IDIs) in the Thai language. This interviewer followed the semi-structured interview guide (Table 1) and asked probing questions to explore the deeper meaning of any responses. Each IDI took between 30 and 60 min, was conducted using an online VDO or phone call, and recorded using a mobile phone (voice recording mode). The number of IDIs depended on the data saturation, with no new theme emerged [19].

### 2.4. Data Analysis

The audio record files were transcribed verbatim (word-by-word) by a research assistant. The interview transcripts were typed in Microsoft Word (Office 365 University Package: Microsoft, Redmond, WA, USA). Text files were imported into a qualitative research software, NVivo (Release 1.3) (QSR International, Victoria, Australia). Codes were created to identify participants while maintaining individual confidentiality and anonymity. An inductive thematic approach was utilized to analyze the data [20,21]. Two authors (AW and TS) read the transcripts twice to familiarize themselves with the data. The interviewer (TS) created initial codes and managed them into thematic maps. Another author (AW), a family physician who was trained and experienced in qualitative research, cross-checked the codes and thematic maps. Both authors then summarized the final themes and sub-themes, and the research team discussed them to reach a consensus and resolve any disagreements. One author (AW) translated the themes and sub-themes and any selected quotations from Thai to English during manuscript writing. All authors discussed and approved the translation.

### 2.5. Ethics Approval

The study protocol was approved by the Human Research Ethics Committee of Walailak University (approval number: WUEC-21-257-01). An author (AW) invited participants to have face-to-face conversations at the clinic or phone calls. Participation in this study was voluntary. Another author (TS) asked for verbal consent prior to each interview.

## 3. Results

A total of 17 patients living with NCDs and four caregivers participated in IDIs. Patients had a mean age of 65.7 ± 11.3 years and had been diagnosed with NCDs a mean of 4.8 ± 1.1 years previously. Over half (*n* = 12, 57.1%) of patients were female. Patients had been diagnosed with at least one of the following conditions: dyslipidemia (*n* = 21), hypertension (*n* = 15), diabetes mellitus (*n* = 6), cancer (*n* = 1), gout (*n* = 1), anxiety (*n* = 1), or ischemic stroke (*n* = 1). Table 2 shows a summary of patient characteristics.

**Figure 1:**
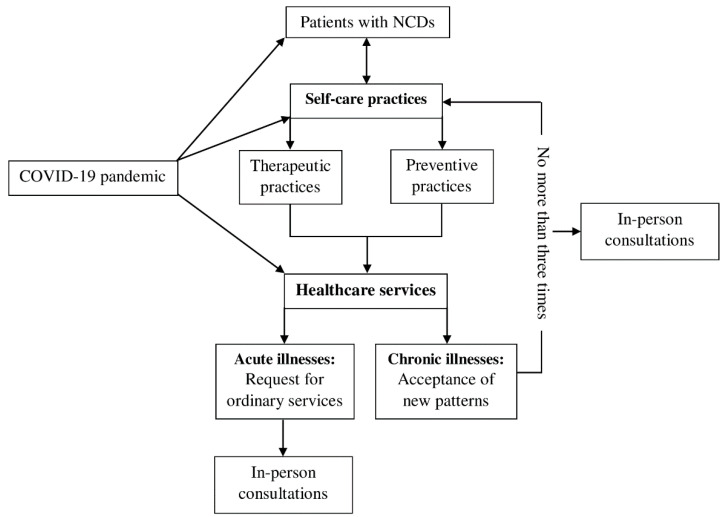
Self-care experiences during the COVID-19 pandemic.

Figure 1 presents a schema of self-care experiences during the COVID-19 pandemic, illustrating the two major themes of experiences among non-communicable diseases (NCDs) patients that emerged from the IDIs: (1) self-care practices during the pandemic and (2) responses to changes in healthcare services.

### 3.1. Self-Care Practices during the Pandemic

Patients and their families were aware of the COVID-19 pandemic and prioritized avoiding activities that would put them at risk of infection. Patients practiced self-care for their underlying illnesses using two different approaches.

#### 3.1.1. Therapeutic Practices

Patients took more medication for their medical conditions than they had previously. Self-care treatments for underlying conditions and non-serious acute illnesses were used to reduce the need for unplanned hospital visits.

For example, one patient said that while she had taken hypertensive medications for four years, she adhered to these prescribed medications and her doctor’s advice more rigidly during the pandemic. If she had any abnormal symptoms, she knew that it would be risky to go to a very crowded place like a hospital and risk infection.

“*I take medicines on time every day. I move and do things carefully. I am very careful. I believe the doctor.” (P9, 62-year-old female pensioner with dyslipidemia and hypertension)*

One patient sought self-medication for a non-serious illness in order to avoid a doctor visit.

“*I started medicines immediately. Used ones bought from a pharmacy. I took them since the symptoms had not been bad. I didn’t need to be in a doctor’s hands. During the COVID situation, if I have any abnormal symptoms, I suddenly take medicines.” (P13, 65-year-old male pensioner with diabetes mellitus and dyslipidemia)*

#### 3.1.2. Preventive Practices

Three key preventive elements, physical activity, diet, and self-hygiene, were addressed by the respondents. Viewpoints were bidirectional based on how the pandemic had impacted the patient or their family and the context of their life.

Healthy habits like engagement in physical activity, healthy diet habits, and personal hygiene were emphasized as primary prevention strategies that patients used to protect themselves from respiratory illnesses, including COVID-19, rather than relying on tertiary prevention (i.e., preventing NCD complications and morbidities).

“*The chronic diseases have been with me for a long time, and I can control them. The doctor can manage them, taking medicines and doing what doctor tells. I just follow the advice, but other diseases can affect daily life, especially COVID. It’s a new disease. I don’t know it. I haven’t experienced and don’t want to get it. I must focus on this disease (COVID-19) by a better self-care action. I have to depend on myself.” (P14, 76-year-old female merchant with dyslipidemia and hypertension)*

A patient who was supported by a government pension, received a regular income every month and had his own house. The COVID-19 pandemic did not affect his financial status and he had private space to engage in activities.

“*People living with chronic diseases are at a higher risk of COVID. I have an underlying disease, so I must protect myself. Luckily, I have monthly income, a house, and the space for exercise. I can exercise without contacting other people. Recently, I do more exercise, more frequent jogging and cycling. I do two times daily in the morning and evening; I wish to be healthier. Safety first! If I got infected, it would be not severe as other high-risk people.” (P4, 64-year-old male pensioner with dyslipidemia and hypertension)*

Another patient said that he had more concerns with self-care and hygiene. His family did not feel comfortable about buying food from the market and they had some free space in the area around their home. They decided to grow vegetables and herbs to boost their immunity against COVID-19.

“*I take care of myself, don’t going out, stay at home, and grow veggies. It’s for good health. I have good food and non-toxic veggies. I grow several veggies such as mugwort, round eggplant, and Fah-Ta-Lai-Jone (Thai name of Andrographis paniculate) that are told as plants for preventing disease.” (P8, 67-year-old male lawyer with diabetes mellitus, dyslipidemia, and hypertension)*

In contrast, a patient who had experienced the social and financial impacts of the COVID-19 pandemic, such as mandatory curfew and fewer customers, felt stress and anxiety. Physical exercise and diet were less important to him. His loss of income reduced his motivation for self-care.

“*Before the COVID, I did exercise, I walked every day together with my friends. Currently, I don’t exercise anymore. I’m afraid of meeting people. I don’t want to go outside. Reduce stress by eating. I’m under a lot of stress and don’t care of whether the food is healthy or not. I eat more but less healthy. Surely, if I see a doctor, my fat and sugar will increase.” (P2, 53-year-old female merchant with anxiety disorder, diabetes mellitus, dyslipidemia, and hypertension)*

### 3.2. Responses to Changes in Healthcare Services

Some patients felt that seeing a doctor was still essential, while other patients and caregivers did not want to visit a location that would put them at high risk of COVID-19 exposure. Some patients needed medical services for their acute illnesses during the pandemic, and preferred to receive in-person care. While all patients accepted the new patterns of healthcare service delivery, which provided remote consultation for their chronic medical conditions, patients also felt that remote services could not totally replace in-person services.

#### 3.2.1. Request for Ordinary Services for Acute Illnesses

Some patients expressed that while they fully understood the situation, they sometimes needed to see a doctor to receive care for acute medical conditions. Healthcare services for non-serious conditions were limited due to the high number of COVID-19 cases in medical settings. If a clinic could not provide services, they were sometimes required to make an appointment with other healthcare institutions or clinics. One patient told clinic staff that his symptoms were more serious than they were to increase his chances of receiving a clinic visit. He talked to staff aggressively to claim that he was a citizen and had the right to receive medical care.

“*I’m not afraid of my underlying diseases, but this disease (COVID-19) disturbs my routine. I used to have ear inflammation. When I have ear pain again, I feel anxious because I’m not sure whether it is COVID or not. I can’t wait, and it is unable to consult by phone. I think I must see a doctor and receive treatments immediately. I called a hospital, but a nurse did not allow me to go. I called several times and told a nurse my pain score was 9; it was 6 actually. She would think it was critical. I told I was unhappy. I had the right to see a doctor, I paid tax. I should get the care. Self-care and wait! It is not right.” (P8, 67-year-old male lawyer with diabetes mellitus, dyslipidemia, and hypertension)*

#### 3.2.2. Acceptance of New Patterns of Care for Chronic Illnesses

Most patients accepted the situation and empathized with healthcare providers. Healthcare sectors had to reduce their workload to provide a safe space for both patients and providers and this led to a new method of consultation. Patients talked with medical staff by phone and received medications by mail. While these approaches were new, patients expressed no concern with the remote services. In addition to telemedicine, self-care began to play a more important role, though one patient reflected that remote services could not replace in-person services in the long term. The patient said that she would only accept remote consultations three times in a row (for appointments occurring every two or three months) before wanting an in-person visit.

“*So many COVID cases, I understand that the doctor postponed the appointment. My diseases are not severe; I just took medicines. They (diseases) are in me, no symptoms, no impacts on my life. The doctor called me and sent me medicines by a postal service, that’s fine. I understand the situation and can accept that. I focus more on self-care, but I don’t want to postpone more than three times (of each 3-month appointment). Three times are almost a whole year. I wish to see a doctor. The doctor can touch me, check my blood, and use his instruments to evaluate any abnormalities. Self-care is a substitute, not a main method.” (P16, 56-year-old female* *government officer with* *dyslipidemia and hypertension)*

## 4. Discussion

This study highlights the response to COVID-19 and the use of self-care practices by patients living with NCDs during the pandemic. Patients with NCDs had disparate perspectives on self-care and used different practices. Patients incorporated self-care practices for therapeutic and preventive purposes such as strict adherence to medications, eating a healthy diet, exercising more regularly, and using self-hygiene. The use of herbal supplements and other forms of self-medication were suggested as options to treat non-severe acute illnesses. The COVID-19 pandemic could be either a facilitator or a barrier to self-care. People from various socioeconomic backgrounds, based on occupation, income, and housing status, were differentially impacted by the pandemic.

Individuals with chronic diseases are particularly vulnerable to COVID-19 and have a higher risk of death, a need for intensive care, and intubation [22,23]. This understanding has increased awareness about the need for self-care among people living with NCDs. Practices of self-care, including adherence to prescribed medications, increasing physical activity, and eating a healthy diet, have varied [10,24,25]. While most patients in this study had improved therapeutic and preventive self-care practices, some patients who were experiencing other difficulties in their lives, such as inadequate income, had increased emotional stress and less motivation to use self-care. The findings of this study illustrated that patients were likely to adhere to prescribed medications and their doctor’s advice. This was necessary for their chronic medical conditions to be appropriately managed [26].

Findings from this study showed that patients became more aware of self-care behavior during the COVID-19 pandemic. However, they still needed access to healthcare services to receive care and medical treatment under the supervision of a healthcare professional. This study reflected the strength of the healthcare system’s ability to provide supportive methods. All study participants were able to receive care for their chronic illnesses in various forms, including through telemedicine and telepharmacy. Moreover, they could also confirm appointments prior to in-person visits or select a remote service. Previous studies showed that >14% of patients missed follow-up visits or prescriptions during the pandemic [10,27,28], and argued that the service system was complex and most patients were afraid to contact COVID-19 when they traveled to see a doctor [10,27]. These studies also suggested that there was a high loss-to-follow-up rate because patients were not confident in the protection provided by the healthcare sector [10,27,28].

Most patients in this study accepted the new pattern of healthcare services and supplemented this with their own self-care practices. All participants still needed medical visits, treatment, and technology which are representative of the whole medical care system [29,30,31]. A possible explanation is that telehealth can provide effective two-way communication between healthcare providers and patients [32], and can serve as a substitute for traditional services. However, some services such as those that require patients and caregivers to play an active role in illness management, may have been altered [32,33]. The absence of human interaction is another limitation of telehealth. Patients may feel less confident in their diagnosis and consultation processes due to a lack of quality time and verbal communication with telehealth services [34].

The findings of this study reflect a deeper understanding of NCD patient self-care and healthcare utilization practices. The lessons learned may apply to the care of all people living with chronic diseases. First, the patients in this study perceived that they were at risk for additional health problems and prioritized self-care practices to maintain their health. Healthcare providers should explore the patterns and rationales of self-care practices for a better understanding of individual’s needs and lived experiences. Healthcare sectors and stakeholders should emphasize the importance of health promotion and disease prevention during and after pandemics. Second, there is an urgent need to develop flexible healthcare that provides both in-person and remote services. Triaging or classifying the needs of healthcare access helps to balance the supply and demand of resources. In addition, the quality and quantity of remote service infrastructure should be improved to assure that healthcare provision is equitable. Moreover, the results reflected that a patient may have a limited threshold for utilizing remote healthcare services. A tailored evaluation of patient’s needs (e.g., phone calls) prior to selecting a remote service or an in-person service may help make the most appropriate decision. Third, this study illustrated the impacts of pandemic COVID-19 on patients through several mechanisms, and there were interactions among individual’s experiences, self-care practices, and healthcare service needs (Figure 1). A comprehensive approach focusing on experiences of an individual patient, self-care practices, and healthcare service needs, is recommended.

This study had some limitations. First, participants were recruited from one clinic. While this limited the generalizability of the study, however, the findings provide a robust understanding of self-care practices in a specific population. Second, all IDIs were conducted through online VDO or telephone calls, which limited the observation of nonverbal communication.

## 5. Conclusions

The COVID-19 pandemic influenced the self-care practices of patients with NCDs. The importance of self-care was considered from a bidirectional perspective. Most patients paid more attention to therapeutic and preventive self-care practices during the pandemic; however, some were too concerned about other aspects of their lives, such as their financial situation. The pandemic led to changes in the pattern of healthcare services. While patients accepted the new methods used to provide services, remote services were not able to replace in-person services in the long term.

## Figures and Tables

**Table 1:** Semi-structured interview guide.

Objectives	Main and Probing Questions
**Impacts of the COVID-19 pandemic on patient’s life and illness**	1. What are the impacts of COVID-19 pandemic on your (or patient’s) life?
	2. What are the impacts of COVID-19 pandemic on your (or patient’s) job/occupation?
	3. What are the impacts of COVID-19 pandemic on your (or patient’s) relationships in the family, community and friends?
	4. What are the impacts of COVID-19 pandemic on your (or patient’s) health?
	5. What are the impacts of COVID-19 pandemic on your (or patient’s) underlying illnesses?
**Patterns and management of life and illness of patient during t** **he COVID-19 pandemic**	1. What do(es) you (or the patient) do as a health prevention method? Why do you (or the patient) do that? What are the differences from the time before the COVID-19 pandemic?
	2. What will you (or the patient) do if you (or the patient) has any other illnesses? Why do you (or the patient) do that? What are the differences from the time before the COVID-19 pandemic?
	3. What do you do with your underlying illnesses? Why do you (or the patient) do that? What are the differences from the time before the COVID-19 pandemic?
	4. Do(es) you (or the patient) have any problems and barriers related to self-care?
	5. What do(es) you (or the patient) need to support self-care more effectively?

**Table 2:** Patient characteristics.

Code	Gender	Age (Years)	Occupation	Underlying Illnesses
**P1**	Male	54	Farmer	Dyslipidaemia and hypertension
**P2**	Female	53	Merchant	Anxiety disorder, diabetes mellitus, dyslipidaemia, and hypertension
**P3**	Female	68	Farmer	Cancer, diabetes mellitus, dyslipidaemia, and hypertension
**P4**	Male	64	Pensioner	Dyslipidaemia and hypertension
**P5**	Female	57	Government officer	Dyslipidaemia
**P6**	Female	62	Pensioner	Diabetes mellitus, dyslipidaemia, and hypertension
**P7**	Female	54	Government officer	Dyslipidaemia
**P8**	Male	67	Lawyer	Diabetes mellitus, dyslipidaemia, and hypertension
**P9**	Female	66	Pensioner	Dyslipidaemia and hypertension
**P10**	Female	62	Pensioner	Dyslipidaemia
**P11**	Male	62	Pensioner	Dyslipidaemia
**P12**	Male	70	Farmer	Dyslipidaemia, gouty arthritis, and hypertension
**P13**	Male	65	Pensioner	Diabetes mellitus and dyslipidaemia
**P14**	Female	76	Merchant	Dyslipidaemia and hypertension
**P15**	Female	51	Government officer	Dyslipidaemia
**P16**	Female	56	Government officer	Dyslipidaemia and hypertension
**P17**	Female	63	Unemployed person with social welfare	Dyslipidaemia and hypertension
P18 *	Female	72	Farmer	Dyslipidaemia and hypertension
P19 *	Male	90	Unemployed person with social welfare	Diabetes mellitus, dyslipidaemia, and hypertension
P20 *	Male	73	Unemployed person with social welfare	Dyslipidaemia and hypertension, and ischemic stroke
P21 *	Male	95	Unemployed person with social welfare	Dyslipidaemia and hypertension

* IDI was conducted with his/her caregiver.

## References

[1] World Health Organization WHO Coronavirus (COVID-19) Dashboard 2021. https://covid19.who.int/.

[2] Moynihan R., Sanders S., Michaleff Z.A., Scott A.M., Clark J., To E.J., Jones M., Kitchener E., Fox M., Johansson M. (2021). Impact of COVID-19 pandemic on utilisation of healthcare services: A systematic review. BMJ Open.

[3] Schnitzler L., Janssen L.M.M., Evers S.M.A.A., Jackson L.J., Paulus A.T.G., Roberts T.E., Pokhilenko I. (2021). The broader societal impacts of COVID-19 and the growing importance of capturing these in health economic analyses. Int. J. Technol. Assess. Health Care.

[4] Aburto J.M., Schöley J., Kashnitsky I., Zhang L., Rahal C., Missov T.I., Mills M.C., Dowd J.B., Kashyap R. (2022). Quantifying impacts of the COVID-19 pandemic through life-expectancy losses: A population-level study of 29 countries. Int. J. Epidemiol..

[5] Saladino V., Algeri D., Auriemma V. (2020). The Psychological and Social Impact of COVID-19: New Perspectives of Well-Being. Front. Psychol..

[6] Wang C., Tee M., Roy A.E., Fardin M.A., Srichokchatchawan W., Habib H.A., Tran B.X., Hussain S., Hoang M.T., Le X.T. (2021). The impact of COVID-19 pandemic on physical and mental health of Asians: A study of seven middle-income countries in Asia. PLoS ONE.

[7] Chiesa V., Antony G., Wismar M., Rechel B. (2021). COVID-19 pandemic: Health impact of staying at home, social distancing and ‘lockdown’ measures—A systematic review of systematic reviews. J. Public Health.

[8] Nikoloski Z., Alqunaibet A.M., Alfawaz R.A., Almudarra S.S., Herbst C.H., El-Saharty S., Alsukait R., Algwizani A. (2021). COVID-19 and non-communicable diseases: Evidence from a systematic literature review. BMC Public Health.

[9] Bello B., Useh U. (2021). COVID-19: Are Non-Communicable Diseases Risk Factors for Its Severity?. Am. J. Health Promot..

[10] Devi R., Goodyear-Smith F., Subramaniam K., McCormack J., Calder A., Parag V., Bizri L.E., Majumdar A., Huang P.H., Bullen C. (2021). The Impact of COVID-19 on the Care of Patients With Noncommunicable Diseases in Low- and Middle-Income Countries: An Online Survey of Patient Perspectives. J. Patient Exp..

[11] Mahmood M.M., Rehman J., Arif B., Rehman Z., Aasim M., Saeed M.T. (2021). Knowledge, attitudes and practices of patients with chronic illnesses during the COVID-19 pandemic: A cross-sectional survey from Pakistan. Chronic Illn..

[12] Palmer K., Monaco A., Kivipelto M., Onder G., Maggi S., Michel J.P., Prieto R., Sykara G., Donde S. (2020). The potential long-term impact of the COVID-19 outbreak on patients with non-communicable diseases in Europe: Consequences for healthy ageing. Aging Clin. Exp. Res..

[13] Gupta S.K., Lakshmi P.V.M., Kaur M., Rastogi A. (2020). Role of self-care in COVID-19 pandemic for people living with comorbidities of diabetes and hypertension. J. Fam. Med. Prim. Care.

[14] Matua G.A., Van Der Wal D.M. (2015). Differentiating between descriptive and interpretive phenomenological research approaches. Nurse Res..

[15] Bradshaw C., Atkinson S., Doody O. (2017). Employing a Qualitative Description Approach in Health Care Research. Glob. Qual. Nurs. Res..

[16] O’Brien B.C., Harris I.B., Beckman T.J., Reed D.A., Cook D.A. (2014). Standards for reporting qualitative research: A synthesis of recommendations. Acad Med..

[17] Tong A., Sainsbury P., Craig J. (2007). Consolidated criteria for reporting qualitative research (COREQ): A 32-item checklist for interviews and focus groups. Int. J. Qual. Health Care.

[18] Patton M.Q. (1999). Enhancing the quality and credibility of qualitative analysis. Health Serv. Res..

[19] Saunders B., Sim J., Kingstone T., Baker S., Waterfield J., Bartlam B., Burroughs H., Jinks C. (2018). Saturation in qualitative research: Exploring its conceptualization and operationalization. Qual. Quant..

[20] Braun V., Clarke V. (2006). Using thematic analysis in psychology. Qual. Res. Psychol..

[21] Gale N.K., Heath G., Cameron E., Rashid S., Redwood S. (2013). Using the framework method for the analysis of qualitative data in multi-disciplinary health research. BMC Med. Res. Methodol..

[22] Hernández-Galdamez D.R., González-Block M.A., Romo-Dueñas D.K., Lima-Morales R., Hernández-Vicente I.A., Lumbreras-Guzmán M., Méndez-Hernández P. (2020). Increased Risk of Hospitalization and Death in Patients with COVID-19 and Pre-existing Noncommunicable Diseases and Modifiable Risk Factors in Mexico. Arch. Med. Res..

[23] Shi S., Qin M., Shen B., Cai Y., Liu T., Yang F., Gong W., Liu X., Liang J., Zhao Q. (2020). Association of cardiac injury with mortality in hospitalized patients with COVID-19 in Wuhan, China. JAMA Cardiol..

[24] De Maria M., Ferro F., Vellone E., Ausili D., Luciani M., Matarese M. (2022). Self-care of patients with multiple chronic conditions and their caregivers during the COVID-19 pandemic: A qualitative descriptive study. J. Adv. Nurs..

[25] Termorshuizen J.D., Watson H.J., Thornton L.M., Borg S., Flatt R.E., MacDermod C.M. (2020). Early impact of COVID-19 on individuals with self-reportedeating disorders: A survey of ~1,000 individuals in the UnitedStates and the Netherlands. Int. J. Eat Disord..

[26] Khayyat S.M., Mohamed M., Khayyat S.S., Alhazmi R.H., Korani M.F., Allugmani E.B., Saleh S.F., Mansouri D.A., Lamfon Q.A., Beshiri O.M. (2019). Association between medication adherence and quality of life of patients with diabetes and hypertension attending primary care clinics: A cross-sectional survey. Qual. Life Res..

[27] Sahoo K.C., Kanungo S., Mahapatra P., Pati S. (2021). Non-communicable diseases care during COVID-19 pandemic: A mixed-method study in Khurda district of Odisha, India. Indian J. Med. Res..

[28] Gummidi B., John O., Jha V. (2020). Continuum of care for non-communicable diseases during COVID-19 pandemic in rural India: A mixed methods study. J. Fam. Med. Prim. Care.

[29] Abraham J. (2010). Pharmaceuticalization of society in context: Theoretical, empirical and health dimensions. Sociology.

[30] Williams S., Martin P., Gabe J. (2011). The pharmaceuticalisation of society? A framework for analysis. Sociol Health Illn..

[31] Bell S.E., Figert A.E. (2012). Medicalization and pharmaceuticalization at the intersections: Looking backward, sideways and forward. Soc. Sci. Med..

[32] Neubeck L., Hansen T., Jaarsma T., Klompstra L., Gallagher R. (2020). Delivering healthcare remotely to cardiovascular patients during COVID-19: A rapid review of the evidence. Eur. J. Cardiovasc. Nurs..

[33] Allemann H., Poli A. (2020). Designing and evaluating information and communication technology-based interventions? Be aware of the needs of older people. Eur. J. Cardiovasc. Nurs..

[34] Shah M.N., Morris D., Jones C.M.C., Gillespie S.M., Nelson D.L., McConnochie K.M., Dozier A. (2013). A Qualitative Evaluation of a Telemedicine-Enhanced Emergency Care Program for Older Adults. J. Am. Geriatr. Soc..

